# A Wise Intervention for Adolescents Who Purchase Loot Boxes (WISEBOx): Pilot Study

**DOI:** 10.2196/87126

**Published:** 2026-11-05

**Authors:** Joaquín González-Cabrera, Vanessa Caba-Machado, Francisco J Sanmartín, Juan Antonio Moriana, Teresa Galán-Luque, Juan Manuel Machimbarrena

**Affiliations:** 1 Instituto de Transferencia e Investigación (ITEI), Universidad Internacional de La Rioja (UNIR), Logroño, Spain; 2 Facultad de Educación, Centro de Formación del Profesorado, Universidad Complutense de Madrid, Madrid, Spain; 3 Departamento de Psicología, Facultad de Ciencias de la Educación y Psicología, Universidad de Córdoba, Córdoba, Spain; 4 Facultad de Psicología, Universidad del País Vasco / Euskal Herriko Unibertsitatea (EHU), Avenida de Tolosa 70, Donostia, 20018, Spain, 34 943 018 344

**Keywords:** loot boxes, wise intervention, gambling, adolescents, video games, microtransactions

## Abstract

In this 1-group pretest-posttest pilot of WISEBOx (Wise Intervention for Adolescents Who Purchase Loot Boxes) with adolescents who had previously purchased loot boxes (N=38), fewer participants reported purchasing loot boxes at the 1-month posttest than in the year before baseline. These findings support the feasibility of WISEBOx and the need for controlled efficacy testing.

## Introduction

Video games are part of mainstream culture and interactive entertainment and represent a major global industry, generating approximately US $187.7B in 2024 [1]. In-game purchases are increasingly common. One prominent format is the loot box (LB): a purchasable virtual item that provides random rewards in exchange for real money [2,3]. LB purchasing has been linked to gambling-related problems [4,5], especially among minors, and to broader harms when consumption becomes problematic [6]. Recent Spanish school-based programs have begun to include LBs within broader multirisk prevention approaches. The DOMINUS program includes a specific LB session and has reported positive indicators for reducing LB purchasing [7]. However, to our knowledge, no validated brief school-based intervention has targeted LB purchasing as its sole focus among adolescents. We therefore designed WISEBOx (Wise Intervention for Adolescents Who Purchase Loot Boxes), a single-session, teacher-delivered wise intervention (WI) [8], and piloted its feasibility and preliminary short-term changes in LB-related behaviors among adolescents who had previously purchased LBs.

## Method

A 1-group pretest-posttest pilot was conducted in two publicly funded Spanish schools with students in the third to fourth year of Compulsory Secondary Education. Participating schools were recruited through professional contacts with school leadership and counseling staff; sampling was nonprobabilistic and convenience based. Of 164 invited students, 65 provided usable paired pre-post data after consent/assent and pseudonymized linkage: 38 with a history of previous LB purchasing (group A) and 27 without a history of LB purchasing (group B). Group A received WISEBOx and constituted the analyzed sample (21/38 [55.3%] were boys; mean age 14.97 [SD 0.79] years; range 14‐16 years). Group B was included to allow whole-class implementation and to ensure that students without LB history received an age-appropriate prevention activity, not to serve as a control group. Participants in group B received a parallel general gambling-prevention session and were not analyzed. WISEBOx is a 70‐ to 80-minute, teacher-delivered indicated-prevention WI based on guided self-reflection, values affirmation, and personal commitment. Four teachers delivered the session after 3 hours of training. A private filter question assigned students to group A or group B without disclosing individual responses. Assessments were generally completed in April 2025 (pretest), at least 1 week before the start of the intervention. The posttest, conducted after the intervention, was administered mainly in late May 2025. Intervention details are shown in Multimedia Appendix 1 and on the WISEBOx website [9]. The primary outcome was LB purchase: a purchase during the past 12 months at pretest and any purchase during follow-up at posttest. Secondary outcomes among posttest buyers included purchase frequency (0 to ≥50), money spent in the last month (none to ≥€100; €1=US $1.14 as of June 3, 2025), LB-related problems (yes/no), and problematic LB use. Problematic LB use was assessed with the Problematic Use of Loot Boxes Questionnaire (PU-LB) [10], an 18-item scale scored from 0 to 5. Mean scores were computed only when all items were valid at both assessments. Materials were developed via iterative qualitative consensus with 6 experts on psychology, education, and responsible gambling using focus groups and reflexive thematic analysis [11].

### Statistical Analysis

Analyses were conducted in SPSS software (version 25; IBM Corp). The McNemar exact test was used for the paired binary primary outcome. Given the nonequivalent pretest and 1-month posttest windows, this test was interpreted as paired discordance rather than as evidence of cessation or efficacy. Wilcoxon signed-rank tests and the Rosenthal r were used for paired ordinal/scale secondary outcomes among available cases. These small denominators did not reflect attrition or data-collection problems but followed from the design itself: secondary outcomes were defined only for the 6 participants who still reported LB purchasing at the 1-month posttest, and requiring complete paired data further reduced available cases to fewer than 10 per outcome. Consequently, these paired comparisons were treated as strictly exploratory.

### Ethical Considerations

This study complied with the Declaration of Helsinki and EU-Spain data protection regulations and was approved by the Research Ethics Committee of the International University of La Rioja (PI067-2024). Written family consent and written adolescent assent were obtained. Participation was voluntary, with two withdrawals before WISEBOx began. Data were pseudonymized for pre-post linkage and stored in a secure, password-protected database accessible only to the research team. No compensation was provided.

## Results

Table 1 summarizes the descriptive statistics and paired tests. At the 1-month posttest, 6/38 (15.8%) adolescents in the analyzed sample reported LB purchasing. The McNemar exact test was significant (*P*<.001), with nonequivalent pretest and 1-month posttest windows. Among posttest buyers, money spent, purchase frequency, and mean problematic LB use were analyzed as exploratory available-case outcomes.

**Table 1.** Descriptive statistics, analytic denominators, and paired tests for loot box (LB)–related outcomes. Prettest LB purchase refers to the previous 12 months, whereas posttest LB purchase refers to the approximately 1-month follow-up. Secondary analyses were restricted to participants who reported LB purchasing at posttest and had complete paired data for each outcome. Purchase frequency and money-spent medians correspond to ordinal response-category codes, not raw numbers of boxes or euros.

Outcome	Pretest	Posttest	Test/result^a^
LB purchase	38/38, 100%	6/38, 15.8%	Exact McNemar: *P*<.001; paired difference=−84.2 percentage points
Purchase frequency among posttest buyers (n=6)	Median 2.00 (IQR 2.00‐4.00)	Median 2.00 (IQR 2.00‐2.00)	Wilcoxon: *Z*=−1.414; *P*=.157; *r*=0.577
Money spent among posttest buyers (n=5)	Median 4.00 (IQR 4.00‐4.00)	Median 2.00 (IQR 2.00‐3.00)	Wilcoxon: *Z*=−2.070; *P*=.038; *r*=0.926
PU-LB^b^ mean score among posttest buyers (n=4)	Mean 2.00 (SD 0.75)	Mean 1.18 (SD 1.58)	Wilcoxon: *Z*=−1.214; *P*=.225; *r*=0.607

a For Wilcoxon signed-rank tests, Rosenthal’s r was calculated as Z/√N and interpreted as an effect-size estimate for exploratory paired comparisons.

b PU-LB: Problematic Use of Loot Boxes Questionnaire.

## Discussion

This pilot addressed the stated aims by suggesting that WISEBOx was deliverable in school settings and that fewer adolescents with previous LB purchasing reported LB purchasing at the 1-month posttest than had reported past-year purchasing at baseline. Secondary available-case outcomes among posttest buyers moved descriptively in the expected direction but remain exploratory (Table 1). These patterns are consistent with the WI rationale of brief values-based reframing [8] and suggest that a concise teacher-delivered module may be feasible for school prevention practice. WISEBOx may complement DOMINUS, a broader Spanish multirisk program that includes an LB session [7], by offering an LB-specific indicated module for adolescents with prior purchasing history. However, these findings are not evidence of efficacy because of the 1-group design, convenience sample, nonequivalent purchase windows, and very small secondary subsamples. The pilot also does not evaluate whether WISEBOx prevents the initiation of LB purchasing among adolescents with no prior LB purchasing history. Feasibility evidence was limited to teacher delivery after brief training and retention of usable paired data; no formal acceptability or fidelity scale was administered. Future research should use adequately powered cluster-randomized trials with equivalent measurement windows, longer follow-up, and formal implementation indicators.

## Supplementary material

10.2196/87126 Multimedia Appendix 1 WISEBOx and parallel gambling-prevention session content and structure.

## Abbreviations

LB: loot box
PU-LB: Problematic Use of Loot Boxes Questionnaire
WI: wise intervention
WISEBOx: Wise Intervention for Adolescents Who Purchase Loot Boxes
