# Supplementary material for: A Wise Intervention for Adolescents Who Purchase Loot Boxes (WISEBOx): Pilot Study
Source: JMIR Form Res. 2026 Nov 5;10:e87126. doi: 10.2196/87126 (PMC13738161; doi:10.2196/87126)
Supplement: Supplementary file 1 [file formative-v10-e87126-s001.docx]

**Multimedia Appendix 1. WISEBOx and Parallel Gambling-Prevention Session Content and Structure**

| **Activity** | **Specific Objective** | **Duration and Group** | **Relevant Methodological Details** |
| --- | --- | --- | --- |
| Introduction | Present the problem, explain the procedure, and divide the class. | 10 minutes (All students) | Brief introduction to the topic and explanation of the LB as a “chest” with randomized rewards that may require spending real money or meeting conditions to open, eliciting emotions similar to gambling. |
| Activity 1: Initial Reflection | Activate self-reflection on the meaning and risks of LBs. | Group A: 20 minutes; Group B: 40 minutes | Open-ended discussion prompts: if an LB constitutes a gamble, what risks does it entail and what emotional payoff does purchasing/opening it provide? In Group B, the reflection begins with gambling in general before defining LB and focuses the entire time on (online) gambling more broadly. |
| Activity 2: Identification and Affirmation of Personal Values | Facilitate the identification of personal values and reflect on potential conflicts with LB use. | 15 minutes  (Group A) | Introspective exercise: list the three most important values (e.g., mental health, self-care, healthy fun) and write how LB use affects or relates to those values. |
| Activity 3: Guided Plenary Discussion | Facilitate group reflection on value–behavior coherence. | 15 minutes  (Group A) | Questions about discrepancies between values and behavior regarding LBs, and about more value-congruent alternatives for spending time/money. The teacher facilitates without imposing conclusions. |
| Activity 4: Alternatives and Personal Commitment | Identify healthy alternatives and reaffirm commitment. | 15 minutes  (All students) | Propose alternatives (e.g., playing with friends, seeking free games). Draft a values-based personal commitment to manage LB use (Group A) or define a future stance/general gambling-prevention commitment (Group B). |
| Activity 5: Closing | Reinforce the commitment and key takeaways. | 5 minutes  (All students) | Emphasize the power of self-reflection and personal control over decisions. |

Note: Group A = primary target group of the intervention: students who have purchased loot boxes and received the full WISEBOx module. Group B = students who have never purchased loot boxes; they received a parallel general gambling-prevention session that shared brief introductory/closing classroom elements but not the LB-specific values-based WISEBOx activities. LB = loot box.
